# The effect of vitamin D status on ovarian reserve markers in infertile women: A prospective cross-sectional study

**DOI:** 10.18502/ijrm.v18i2.6501

**Published:** 2020-02-27

**Authors:** Nazanin Alavi, Mahbod Ebrahimi, Firoozeh Akbari-Asbagh

**Affiliations:** Obstetrics and Gynecology Ward, Yas Hospital, Tehran University of Medical Sciences, Tehran, Iran.

**Keywords:** Anti-Müllerian hormone, Infertility, Vitamin D deficiency, Ovarian follicle.

## Abstract

**Background:**

Vitamin D deficiency and infertility are two important health problems in Iran. Some studies suggest that vitamin D may influence Anti-Müllerian hormone (AMH) and antral follicle count (AFC) as an ovarian reserve.

**Objective:**

The present study aimed to investigate the impact of vitamin D on AMH serum concentrations/AFC.

**Materials and Methods:**

Three hundred and five infertile women referred to the IVF Unit of Yas hospital, between July and December 2017, were enrolled in this cross-sectional study. The demographic characteristics of the participants, as well as the serum levels of vitamin D, AMH, and ultrasonic examination of AFC were recorded.

**Results:**

Finally, 287 infertile women were included in the analysis with a mean age of 29.95 ± 4.73 yr (18-45 yr) and a mean Body mass indexof 25.11 ± 4.41 kg/m${}^{2}$. The median AMH and vitamin D levels were 3.20 and 22.82 ng/ml, respectively. Considering the cut-off level of 20 ng/ml, 58.7% were vitamin D deficient. Regression analysis showed no association between AMH and vitamin D levels (p = 0.161), even after adjusting for baseline variables (p = 0.182). A total of 120 patients had an AFC < 6 and 164 ≥ 6, which was not statistically different between the groups with normal level or deficient vitamin D (p = 0.133).

**Conclusion:**

The present cross-sectional study showed no significant association between serum levels of vitamin D and AMH or AFC in infertile women, even after adjusting for baseline variables.

## 1. Introduction

Vitamin D, a fat-soluble vitamin is absorbed by the sunlight and considered as a sunshine hormone. It has significant roles in different parts of the body, suggested to be due to the expression of vitamin D receptor (VDR) on different organs. Hence, it plays a significant role in insulin resistance (1), cancers, and a variety of chronic diseases (2, 3). Despite appropriate sun light supply in Iran, a high prevalence of vitamin D hypovitaminosis was reported in the Iranian population, especially in women (4).

As a result, VDR is also expressed on the male and female reproductive systems, studies suggest the role of vitamin D in infertility (5), with a lifetime prevalence of about 25% (6). Several studies have thus addressed the association of vitamin D with different markers indicating infertility (7, 8). Ovarian reserve can be measured by antral follicle count (AFC), but it requires ultrasound examination and may be biased (9). Anti-Müllerian hormone (AMH), a member of the TGF-beta (transforming growth factor-beta) superfamily of proteins, which produced by granulosa cells and secreted by pre-antral and antral follicles (10), has been suggested as a better index of ovarian reserve of follicles than AFC (11) and is mainly used in assisted reproductive technology. It has been suggested that vitamin D changes AMH signaling by downregulation the expression of AMH receptor-II gene (12), follicle-stimulating hormone (FSH) sensitivity and progesterone production. Moreover it has an important role in follicular development, differentiation, and luteinization (13).

Although the association of vitamin D and AMH levels have been proposed by animal studies (14, 15), clinical human studies failed to prove such an association (16, 17) and some have reported variable association based on season or sex (18). In addition, various studies have studied the association of vitamin D and AMH with different causes of infertility, like endometriosis and polycystic ovarian syndrome (PCOS) (19) and have shown the significant role of vitamin D, while other studies have suggested no association (20).

Due to the inconsistency of the results of studies testing this hypothesis and the significance of hypovitaminosis D in Iran, the present study aimed to investigate the association between vitamin D and AMH/AFC on an Iranian population.

## 2. Materials and Methods

### Study design

The present prospective cross-sectional study investigated all infertile women referred to the IVF Unit of YAS Hospital from July to December 2017 for fertility evaluation.

The sample size of this study was calculated at 305, based on the study of Drakopoulos and colleagues (16), considering α = 0.05 and power of 80%. Patients who met the following inclusion criteria were enrolled into the study by convenient sampling method until saturation of the sample size: women, aged 18 to 45 yr, diagnosed with infertility by the gynecologist, defined as no conception after 12 months of no contraceptive methods, who did not use any vitamin supplements, and gave written consent for participation into this study. Women with any underlying endocrine or metabolic disease were excluded from the study. Also, any patient who refused to continue the study or did not complete the study was excluded.

After confirmation of the diagnosis, the characteristics of all eligible patients were recorded in checklists designed for this study. The recorded variables included age, weight, and height (for calculation of body mass index [BMI]), etiology of infertility, and smoking status. The measurement of patients' height and weight were performed by standard protocols in the same clinic and recorded in the checklist.

Then, patients were referred for further serum laboratory tests and ultrasound examination. One non-fasting blood sample was taken from patients from the left hand's cubital vein in the sitting position that was sent to the laboratory of the hospital. The samples were centrifuged and stored at 20ºC. Vitamin D status was measured by assessing serum levels of 25 OH-D in frozen samples using enzyme immune assay (ELISA, Accu-Bind, Monobind, Inc, Lake Forest, USA). The sensitivity of the 25 OH-D vitamin D Accu-Bind ELISA test system is 0.67ng/ml. The intraassay and interassay variation coefficients were 3.95 % and 5.62%, respectively. The AMH was measured by the ELC method using Roche device (Cobas E411, made in Germany). AFC was measured in early follicular phase.

Then, patients were referred to the radiology clinic of the hospital for a vaginal ultrasound. All ultrasound examinations were performed by one radiologist, who counted the follicles with 2_9 mm diameters and recorded them as AFC.

The results of laboratory and ultrasound measurements were recorded in the checklist and used for analysis. After data collection, the patients were scheduled for the treatment plan, based on the gynecologists' opinion, which was recorded in the checklist and used for analysis.

### Ethical consideration

The study was approved by the Ethics Committee of Tehran University of Medical Sciences (Code: IR.TUMS.MEDECINE.1396-3430). Before recruitment, the design and objectives of the study were explained to all participants and those who were willing to participate in the study signed the written informed consent. No additional costs were imposed on the patients and all ethical principles of the latest version of the Declaration of Helsinki on human studies were met throughout the study.

### Statistical analysis

The results of quantitative variables were reported by the median, mean ± standard deviation (SD), and categorical variables by frequency (percentage). Kolmogorov-Smirnov test was used to assess the normal distribution of data, the results of which indicated that distribution of AMH and Vitamin D levels were not normal, therefore nonparametric tests, such as Mann-Whitney U-test, were used for their analysis. Other continuous variables were compared using *T*-test. Categorical variables were compared using the chi-square test. For a better comparison of variables with vitamin D levels, this continuous variable was considered as two categories (normal level or deficient) based on the cut-off point of 20 ng/ml (21). The association of variables was tested by Pearson's correlation coefficient and multiple regression model. For all statistical analysis, the statistical software IBM SPSS Statistics for Windows version 21.0 (IBM Corp. 2012. Armonk, NY: IBM Corp) was used. P-values < 0.05 were considered statistically significant.

## 3. Results

A total of 305 infertile women were included in the study. Eighteen patients did not complete the study. Finally, 287 infertile women were included in the analysis. Patients' age ranged from 18- 45 yr with a mean ± SD of 29.95 ± 4.73 yr. The demographic and clinical characteristics of participants are shown in table I. Median AMH and vitamin D levels are also shown in table I. Considering the cut-off level of 20 ng/ml, patients were categorized into two groups and demographics and AMH levels were compared between the groups. As shown in table I, 58.7% were vitamin D deficient and the rest were not, and there were no difference between the groups in terms of age, BMI, and AMH levels. Also, serum vitamin D levels were not different in Summer compared to Autumn (p > 0.05) (Table I).

**Figure 1:**
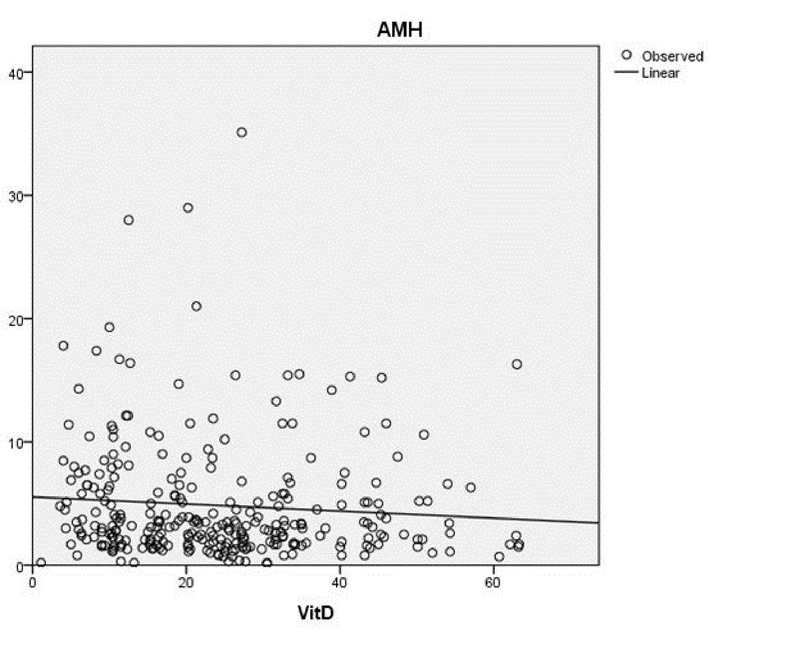
Correlations plot between 25-OH vitamin D and anti-Müllerian hormone (AMH).

The AFC levels (categorized as < 6 and ≥ 6) are compared with AMH and vitamin D sufficiency in table II. As demonstrated, there was no statistically significant difference between the groups with normal level or deficient vitamin D, while median AMH levels were significantly higher in AFC levels ≥ 6 (p < 0.001). Regression analysis showed no statistically significant association between AMH and vitamin D levels (Spearman's r = -0.083, 95% CI:-0.69-0.011, p = 0.161) (Figure 1). After adjusting for potential confounders (age and BMI), the results of multiple linear regression analysis showed no association between serum levels of vitamin D and AMH levels (Spearman's r = -0.080, p = 0.182).

**Table 1:** Comparison of demographic and clinical characteristics of participants according to vitamin D sufficiency


**Variable**		**Total**	**Vitamin D ng/ml**		**P-value**
		**> 20**	**≤ 20**	
Age (yr)*		29.95 ± 4.73	30.14 ± 4.59	29.68 ± 4.96	0.551${}^{†}$
BMI (Kg/m${}^{2}$)*		25.11 ± 4.41	25.11 ± 4.18	25.10 ± 4.10	0.936${}^{†}$
Season**					
	**Summer**	137 (47.7%)	79 (27.5%)	58 (20.2%)	
	**Autumn**	150 (52.3)	87 (30.3%)	63 (21.9%)	0.819${}^{§}$
AMH, ng/ml***		3.20	2.70	3.80		0.93${}^{‡}$
*Data presented as Mean ± SD **Data presented as n (%) ****Data presented as median ${}^{†}$ *t *test ${}^{‡}$Chi-square ${}^{§}$ANOVA BMI: Body mass index AMH: Anti-Müllerian hormone					

**Table 2:** Comparison of AFC value according to median anti-Müllerian hormone and the frequency of the studied patients in different categories of 25-OH vitamin D


**Category**	**Total count**	**Median AMH**	**P-value** ${}^{†}$	**Vitamin D > 20 ng/ml, No**	**Vitamin D < 20 ng/ml, No**	**P-value** ${}^{†}$
**AFC < 6**	120	1.62	74	46	
**AFC ≥ 6**	164	4.26	< 0.001	89	75	0.133
${}^{†}$The results of ANOVA AFC: Antral follicle count AMH: Anti-Müllerian hormone						

## 4. Discussion 

The results of the present study revealed that a majority of our patients were in vitamin D deficiency zone (< 20 ng/ml). The median vitamin D level was close to the cut- off of deficiency.These results are consistent in line with the results of previous studies, indicating a high prevalence of vitamin D deficiency in the Iranian population, especially women (22-24). Another study in Tehran reported that 54% of women living in Tehran had a serum vitamin D level of < 10 ng/ml and 32% of them had a serum vitamin D level of 10-20 ng/ml (22). One study on reproductive age women who were residences of Tehran, has also shown that more than 85% had a serum vitamin D level of less than 20 ng/ml (25). Although the prevalence rate in the aforementioned studies are higher than that of ours, their results suggest a high prevalence of vitamin D deficiency in this population, which confirms the results of our study. This is while Tehran, located in 36°21”N, has a mean sun exposure of 8 hr / day. However, women are mainly stay indoors (23). In addition, air pollution is considered one of the causes of the high prevalence of vitamin D deficiency in women living in Tehran (22).

Considering the fact that vitamin D deficiency is associated with several diseases and can predispose patients to some diseases or deteriorate the disease status, it is important to pay attention to vitamin D levels of patients (26). Infertility is one of the diseases associated with vitamin D deficiency and women with infertility are expected to have a higher prevalence of vitamin D deficiency, which emphasizes the significance of paying attention to the serum level of vitamin D in infertile women and appropriate supplementation of deficient cases (27, 28). Therefore, in the present study, we investigated the association of vitamin D deficiency with their ovarian reserve in infertile women. In our study, AMH was recorded in 287 infertile women, who were referred to a specialized infertility clinic and the results showed a median AMH level of 3.20 ng/ml, significantly associated with lower AFC levels. This association suggests the value of AMH in predicting follicle count, as a simpler test, which confirms the results of previous studies (29, 30).

For testing the main hypothesis of the present study, the association of AMH and AFC levels with vitamin D levels were tested. The results of our analysis indicated that neither AMH nor AFC levels differed between groups with deficient or sufficient vitamin D levels. Also, regression analysis showed no association between AMH and vitamin D levels. These results suggested that serum levels of vitamin D were not related to AMH levels. This hypothesis has been evaluated by few studies previously. Pearce and colleagues, in their study investigated the association of AMH with vitamin D in 340 women (58 with PCOS and 282 ovulatory women) who aged below 40 yr; the results of this study reported no difference in the serum level of vitamin D between the two groups and indicated no association between AMH and vitamin D levels in neither groups (17). These results are similar to that of our study, suggesting that the serum levels of vitamin D are not associated with AMH; although we evaluated infertile women with any cause. As AMH is important in pathophysiology of PCOS (19), the results of our study are comparable to those studying PCOS (13, 19). The literature review also suggests that vitamin D supplementation can neither improve AMH production in women with PCOS (13) nor does it reduce the risk of endometriosis (31). Molecular investigation has also showed that VDR polymorphism did not cause endometriosis or infertility (20). These results confirm the general conclusion of our study, indicating no association between vitamin D and infertility, although in our study, we did not examine the cause of infertility and nor did we separate the results according to the cause of infertility. In addition to the BMI and seasonal changes, which we considered for adjusting the results accordingly, there are several other factors that can affect the serum levels of vitamin D.

As far as we are concerned, there are very few studies that reported a positive association between AMH and serum levels of vitamin D. In the study by Dennis *et al*., 113 adult men, 33 women of reproductive age, and 74 young boys were investigated, and the results showed that vitamin D is a regulator of AMH in men and women but not in boys and recorded a positive association between AMH and serum levels of vitamin D (18). However, this study had some methodological problems such as small sample size. Therefore, the results may not be conclusive. In another Iranian study, 30 infertile women were treated with 50,000 IU weekly vitamin D supplements for three months and the results showed a significant increase in AMH and vitamin D levels after treatment; according to the results of this study, AMH levels increased from 0.513 ng/ml before treatment to 1.048 ng/ml after treatment in women with vitamin D deficiency (32). Although they reported increased AMH, the post-treatment AMH levels were still below the lower limit of AMH (< 1.5 ng/ml) (31), while in the present study, the median AMH of our participants was within the normal range. Therefore, the difference in the clinical characteristic of patients could be the underlying factor for the differences between studies. Furthermore, the discrepancy among the results of studies might be due to the seasonal changes in vitamin D values, different measurement techniques, and different causes of infertility that can cause differences in vitamin D levels.

Although the present study investigated the association of AMH and FSH with serum levels of vitamin D in a sufficient sample of infertile women, this study could have several limitations. Firstly, the cross-sectional nature of the study that limited the generalizability of the results and reporting the short- and long-term follow-up results of participants. Secondly, there are a wide range of variables that can affect the serum level of vitamin D, as well as AMH levels, which can affect the results of this study. Although the cause of infertility may change the serum levels of vitamin D, it was not within the study objectives and its assessment requires further studies. Meanwhile, we evaluated all samples with one kit and using one device to reduce the confounding effect of different measurement techniques on serum levels of vitamin D and one radiologist interpreted all AFC levels, performed in one center, to reduce the confounding effect of these variables. Finally, we adjusted the results for BMI and seasonal changes in serum levels of vitamin D.

## 5. Conclusion

In conclusion, the present cross-sectional study showed a high prevalence of vitamin D deficiency in the sample of 287 infertile women, referred for treatment; however, there was no significant association between the serum levels of vitamin D and AMH or AFC in infertile women, even after adjusting for confounders. Studying other indices of reproduction/ovarian reserve and controlling the effect of confounders in future randomized clinical trials can investigate this hypothesis more accurately and suggest the therapeutic efficacy of vitamin D supplementation on infertility.

##  Conflicts of Interest 

There is no Conflicts of Interest
